# Do eye movements in REM sleep play a role in overnight emotional processing?

**DOI:** 10.1016/j.neuropsychologia.2025.109169

**Published:** 2025-05-12

**Authors:** Kyrillos M. Meshreky, Penelope A. Lewis

**Affiliations:** School of Psychology, Cardiff University, Cardiff, UK

**Keywords:** REM sleep, Eye movements, REM density, Emotional processing, Superior colliculus, EMDR, Amygdala

## Abstract

Eye movements (EMs) are a defining feature of rapid eye movement (REM) sleep, yet we are still not clear why they happen. A few hypotheses attempt to explain the possible underlying mechanisms. However, a clear understanding of their functional significance remains lacking. Interestingly, there is an EM component in Eye Movement Desensitization and Reprocessing (EMDR) therapy, that is approved for post-traumatic stress disorder (PTSD). The developer of EMDR, Francine Shapiro described the technique as mimicry of REM. Robert Stickgold built on this by proposing a putative neurobiological model in which the repeated eye movements in EMDR initiate brainstem REM-like mechanisms. In this article, we combine Stickgold’s model with the results of a study which showed that alternating bilateral visual stimulation in mice yielded sustained increases in the activities of the Superior Colliculus (SC) and mediodorsal thalamus (MD) which suppressed the activity of basolateral amygdala. We pose a hypothetical question: could EMs during REM sleep similarly inhibit amygdala activity through the SC-MD pathway? And could this be part of the affective modulation mechanisms characteristic of REM sleep?

## 1 Introduction

Recurrent periods of rapid eye movements (EMs), along with the reduction of prior high-voltage activity of non-rapid eye movement (NREM) sleep, were first recorded as features of REM sleep in 1953 by (Aserinsky and Kleitman, 1953). Since then, the eye movement phenomenon has become one of the defining characteristics of adult mammalian REM sleep. More recently, EMs have been used to distinguish between two REM microstates: phasic REM, characterized by bursts or trains of EMs, and tonic REM, characterized by the relative absence of EMs or isolated EMs.

Despite being a hallmark of REM sleep, the functional significance of EMs remains a mystery. Could EMs be nothing more than gaze shifts in the virtual world of REM dreams, as suggested by the scanning hypothesis? Are they a mere epiphenomenon of a functional brain activity during REM, such as the ponto-geniculo-occipital (PGO) waves, without being functional themselves? Or could they potentially have a distinct role in inducing functional patterns of brain activity during REM? Another intriguing possibility could be that EMs, initially a byproduct of PGO waves, have evolved over time to play a functional role in modulating brain activity.

### 1.1 Link to REM sleep functions

Findings from sleep studies suggest that EMs during REM may be linked to REM sleep functions. For example, EMs during REM have been associated with learning-related increases in theta power during phasic REM sleep (van den Berg et al., 2023). Specifically, theta power time-locked to EMs in phasic REM sleep was positively correlated with overnight performance improvement in the Tower of Hanoi problem-solving task, strengthening the link between sleep-induced benefits in problem-solving, REM sleep theta, and EMs during REM. This aligns with the view that PGO waves coupled with EMs and theta waves during REM sleep are involved in learning and memory functions.

### 1.2 REM density and emotional processing

Moreover, intensification of phasic REM components, including the average number of rapid EMs per minute in REM sleep (conventionally referred to as REM density), appears to be a marker of affect intensity and emotional processing. This is particularly evident in major depressive disorder (MDD) and post-traumatic stress disorder (PTSD).

In MDD, increased REM density is a prominent feature, along with other signs of REM sleep disinhibition, such as shortened REM latency and prolonged first REM periods. This heightened REM density is not only characteristic of MDD but is also associated with clinical outcomes (Mendoza Alvarez et al., 2025). Interestingly, MDD patients show reductions in REM density during remission, whether through antide-pressants or psychotherapy. Antidepressants are known to inhibit REM sleep by preventing the reuptake of monoamines (serotonin and norepinephrine), creating a state of monoaminergic abundance that suppresses most components of REM sleep. It remains unknown how much of their antidepressant effects may be related to their REM-inhibiting properties.

Similarly, In PTSD patients, increased density of EMs in REM is also among the consistently reported findings (Kobayashi et al., 2007). Pre-sleep exposure to emotional stimuli as seen in trauma-analogue experiments was found to affect REM sleep components with REM-sleep disinhibition. Increased REM density has been proposed as a marker of hypofrontality in MDD and poor emotional processing in MDD/PTSD (Mendoza Alvarez et al., 2025).

Moreover, EMs during REM were found to be reduced but still present in congenitally blind individuals, despite their lack of visual experiences and the potential absence of visual dream imagery (Christensen et al., 2019). Overall, the above findings suggest that EMs during REM sleep may be more than gaze shifts in our dream world. Instead, they could be playing a role in the neural processes of REM sleep and underpin its known functions. These functions include emotional processing (Walker, 2011), integrating newly learned information with pre-existing knowledge (Diekelmann and Born, 2010) and exploring novel associations between weakly connected networks, as proposed by the Network Exploration to Understand Possibilities (NEXTUP) model (Zadra and Stickgold, 2021).

### 1.3 Memory processing during sleep

Despite the lack of a clear mechanistic understanding of how REM physiology facilitates these functions, hypothetical models attempt to build on the current knowledge to provide a coherent explanation. Several models have been proposed to elucidate the distinct roles of sleep stages in the processing of emotional memories and how disruptions in these processes can lead to conditions such as depression and PTSD (Goldstein and Walker, 2014). The ’Sleep to Forget and Sleep to Remember’ model posits that sleep facilitates the decoupling of memory from its associated affect. According to this model, memory reactivation during NREM sleep, particularly slow-wave sleep (SWS), consolidates the memory. Meanwhile, REM sleep attenuates the emotional component of the memory by providing a period during which the amygdala-hippocampal networks are activated while adrenergic activity in the locus coeruleus is reduced (Walker and van der Helm, 2009).

In this article, we will focus on another model, the sleep-dependent transfer and integration of episodic memories described by Stickgold (2002). The model is based on the previous understanding that episodic memories are hippocampus-dependent, as they are initially stored in the hippocampus as a set of ’pointers’ encoding memory-specific activation signatures of the other brain areas initially activated by the memory. These include the sensory cortices, which store the trace of perceptual representation, and the limbic system, which stores the emotional component. Over time, and in a sleep-dependent process, semantic information is extracted from the hippocampal episodic memories and stored in the neocortex, becoming hippocampus-independent semantic neocortical memories. Stickgold proposes that this process may explain how traumatic memories feel less personal and more semantic over time.

Since sensory input is shut down during sleep, it provides an excellent opportunity for the transfer and integration of hippocampal episodic memories into neocortical semantic memories. According to the model, REM and non-REM sleep serve related but distinct sequential functions in off-line memory reprocessing. The relatively higher levels of serotonin and norepinephrine in non-REM sleep allow for the strengthening of hippocampal memories. In contrast, the diminished levels of serotonin and norepinephrine, along with high levels of acetylcholine in REM sleep, allow for the deactivation of the hippocampus while strengthening neocortical memories and preferentially activating weakly associated neocortical networks.

This sequential memory reprocessing in NREM and REM facilitates the integration of episodic memories into semantic memories. This integration through the cortico-hippocampal circuits induces feedback weakening of the episodic memory and its associated affect in the amygdala, allowing both the episodic memory trace and its associated affect to fade. This is particularly important for traumatic memories characterized by heightened affective responses. The memory processing mode that the brain shifts to during REM sleep is believed to play a key role in the weakening of traumatic episodic memories, integrating them into semantic knowledge while uncoupling their affective component. The physiological mechanisms underlying these REM effects likely involve prominent REM brainstem mechanisms, particularly the PGO waves, which propagate to different brain regions, modulating their activities during REM sleep. We still lack a mechanistic understanding of how PGO waves may influence the weakening of hippocampal memories or induce affective uncoupling, leaving the story incomplete.

### 1.4 Directed EMs during wakefulness

In our endeavour to complete the story, we will explore a different yet pertinent line of thought that focuses on the effects of directed EMs during wakefulness on brain activity. These studies have garnered interest since the approval of Eye Movement Desensitization and Reprocessing (EMDR) therapy for the treatment of PTSD, aiming to investigate its mechanism of action. Whether awake directed EMs of EMDR exhibit the same motor characteristics and underlying control mechanisms as the EMs of REM sleep is a subject for debate. However, Shapiro originally described the EMs in her technique as mimicry of REM, and Stickgold (2002) proposed a neurobiological model suggesting that the repeated EMs in EMDR may “push-start” brainstem REM-induction mechanisms. Stickgold’s model also links EMs during REM and EMs during wakefulness as similar brain areas are activated in both (Chong-Hwa Hong et al., 1995).

Notably, studies have found that awake EMs can induce changes in brain activity including suppression of amygdala activity (Macé et al., 2018). In a fear conditioning paradigm, goal-directed EMs were found to enhance extinction learning by suppressing the amygdala and altering its functional connectivity with the dorsal frontoparietal network and the ventromedial prefrontal cortex (de Voogd et al., 2018). Moreover, bilateral EMs effectively reduced recognition memory and affective responses (subjective ratings and psychophysiological responses, including skin conductance response and pupil size) to trauma-related memories (Xu et al., 2023).

Interestingly, Baek et al. (2019) investigated this in mice and found that alternating bilateral visual stimulation induced a lasting reduction of fear during extinction and also identified a neuronal pathway responsible for this effect, between the superior colliculus (SC) and mediodorsal thalamus (MD). This pathway, via a feedforward inhibitory circuit, suppresses the activity of fear-encoding cells in the basolateral amygdala. Optogenetic manipulation of the SC–MD circuit showed that this circuit was both essential and sufficient to persistently suppress fear. Baek et al. (2019) propose this as the mechanism by which bilateral visual stimulation mediates persistent fear extinction in mice.

### 1.5 Superior colliculus (SC)

The SC is a midbrain structure that is well known for its crucial role in controlling EMs during wakefulness and reorienting response. However, its role in REM sleep is less prominently studied. PGO waves are considered the hallmark of REM. They are linked to the direction of EMs during REM and are phase-locked with hippocampal and neocortical theta waves during REM. The neuronal circuits generating PGO waves are located, as the name implies, in the pons (P), lateral geniculate (G), and occipital cortex (O). However, other brain areas, including the SC, are also activated by PGO waves during REM sleep (Nelson et al., 1983). Moreover, REM-locked activation of SC has been reported in a whole night fMRI study (Hong et al., 2009).

**Fig. 1:**
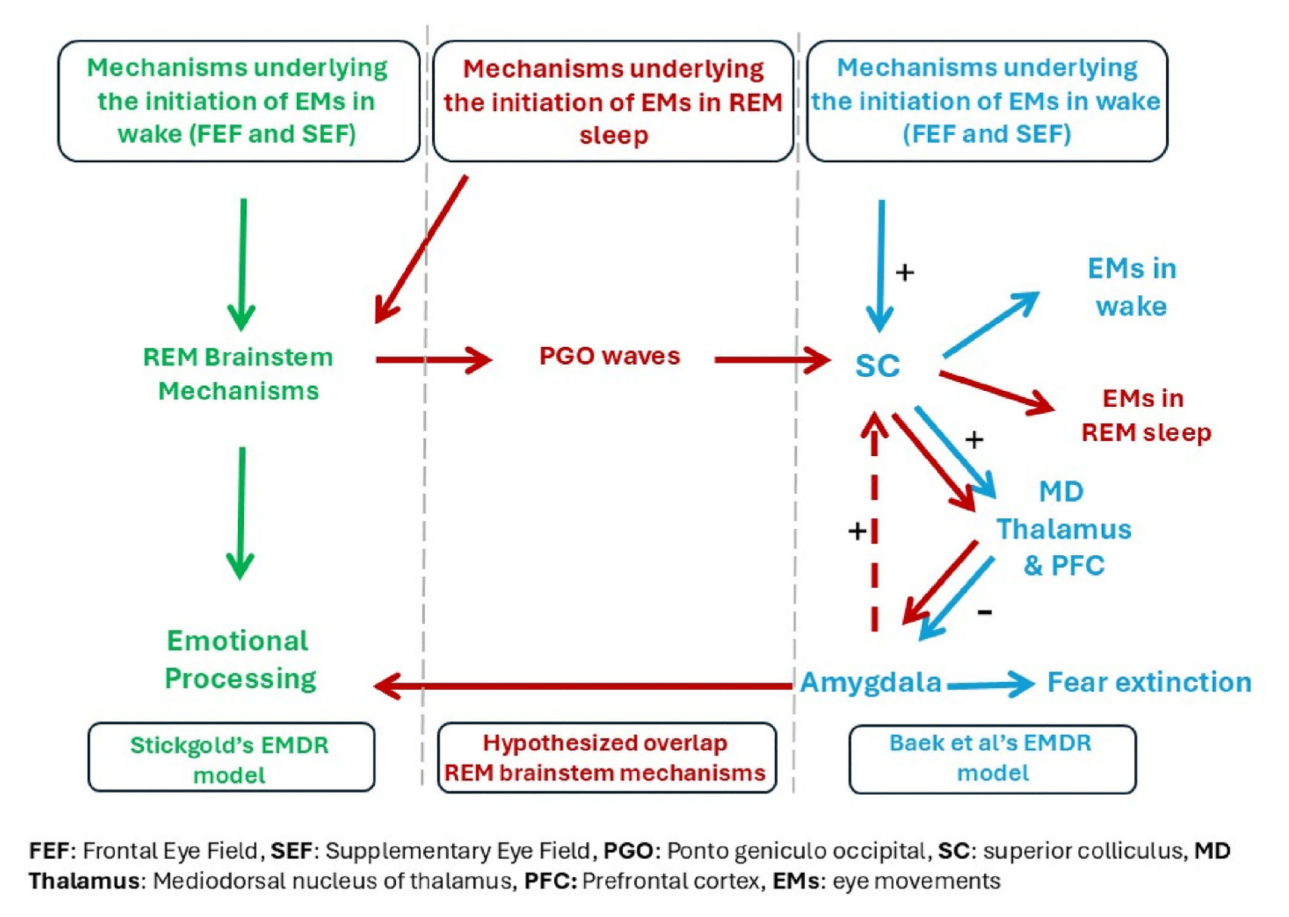
The green pathway (left) represents Stickgold’s hypothesis that EMs in wake initiate REM-like brainstem mechanisms that facilitate emotional processing (Stickgold, 2002). The blue pathway (right) shows the findings from Baek et al. (2019), indicating that alternating bilateral visual stimulation results in the inhibition of the amygdala. The red pathway (middle) illustrates the hypothesized overlap. The red dashed arrow represents a hypothesized feedback circuit between the superior colliculus and the amygdala.

This may suggest that the SC of the midbrain may be activated by the adjacent pontine generators of PGO waves to initiate the EM phenomenon of REM. If this is true, and the SC plays a role in REM EMs, in a manner similar to directed EMs during wakefulness, there may be a reason to think that the SC-MD pathway described by Baek et al. is active during REM. This pathway (or perhaps a more complex equivalent circuit in humans with input from prefrontal cortex) could possibly be a mechanism by which the EM-associated SC activation during REM exerts control over the amygdala. Given that amygdala is known to be hyperactive in REM this could facilitate the reset of its responses overnight, uncoupling amygdala responses from traumatic memories (Fig. 1).

With the SC-amygdala circuit in mind, we may also hypothesize that the level of amygdala activity during REM sleep, which is proportional to the affective component of replayed memories, is fed back to the superior colliculus (SC). This feedback modulates the SC’s activity and, consequently, the frequency of REM EMs to the level required to reduce amygdala activity for this memory overnight. Therefore, it makes sense that traumatic and stressful memories before sleep are associated with an increased frequency of EMs during sleep (Fig. 1). Nevertheless, the latter hypothesis should be taken with caution considering the potential differences in the mechanisms regulating awake EMs and EMs during REM sleep.

### 1.6 Considerations

This article explores the potential mechanisms underlying Stickgold’s REM-induced weakening of the episodic memory and its associated affect, in light of the intriguing and relevant findings from Baek et al. (2019). However, several considerations need to be acknowledged while making this connection. Firstly, the challenge of translating results from rodent studies to humans given the significant structural and functional differences. However, it is difficult to ignore that much of our current knowledge about sleep originally came from landmark animal studies. Secondly, the existence of the SC-MD pathway in humans needs to be further replicated in future studies. While a colliculus-pulvinar-amygdala pathway that encodes the intensity of emotional responses to negative stimuli has been identified in (Kragel et al., 2021), no subcortical pathway from the SC to the amygdala has been well characterized in humans. Thirdly, the activity of the rodent SC-MD pathway was examined during awake EMs. However, it is unclear whether the same would apply to EMs during REM sleep, especially since the similarity of the control mechanisms underlying awake EMs and EMs in REM sleep is a subject of debate. Lastly, it is unclear how the hypothesized mechanisms may differ between depression and PTSD patients and non-clinical populations. Further studies are required to investigate these hypotheses in greater depth.

## Data Availability

No data was used for the research described in the article.
